# Bis(μ-2-carboxymethyl-2-hydroxy­butane­dioato)bis­[diaqua­manganese(II)]–1,2-bis­(pyridin-4-yl)ethane–water (1/1/2)

**DOI:** 10.1107/S1600536812032771

**Published:** 2012-07-25

**Authors:** In Hong Hwang, Pan-Gi Kim, Cheal Kim, Youngmee Kim

**Affiliations:** a Department of Fine Chemistry, Seoul National University of Science & Technology, Seoul 139-743, Republic of Korea; b Department of Forest & Environment Resources, Kyungpook National University, Sangju 742-711, Republic of Korea; c Department of Chemistry and Nano Science, Ewha Womans University, Seoul 120-750, Republic of Korea

## Abstract

The asymmetric unit of the title compound, [Mn_2_(C_6_H_6_O_7_)_2_(H_2_O)_4_]·C_12_H_12_N_2_·2H_2_O, comprises half of a centrosymmetric dimer, half of a 1,2-bis­(pyridin-4-yl)ethane and one water mol­ecule. Two citrate ligands bridge two Mn^II^ ions, the Mn^II^ ion being coordinated by four O atoms from the citrate(2−) ligands and two water O atoms, forming a distorted octa­hedral environment. In the crystal, O—H⋯O hydrogen bonds link the centrosymmetric dimers and lattice water mol­ecules into a three-dimensional structure which is further stabilized by inter­molecular π–π inter­actions [centroid–centroid distance = 3.792 (2) Å].

## Related literature

For inter­actions of metal ions with biologically active mol­ecules, see: Daniele *et al.* (2008 ▶); Parkin (2004 ▶); Tshuva & Lippard (2004 ▶); Stoumpos *et al.* (2009 ▶). For related complexes, see: Lee *et al.* (2008 ▶); Park *et al.* (2008 ▶); Shin *et al.* (2009 ▶); Song *et al.* (2009 ▶); Yu *et al.* (2008 ▶, 2009 ▶); Kim *et al.* (2011 ▶). 
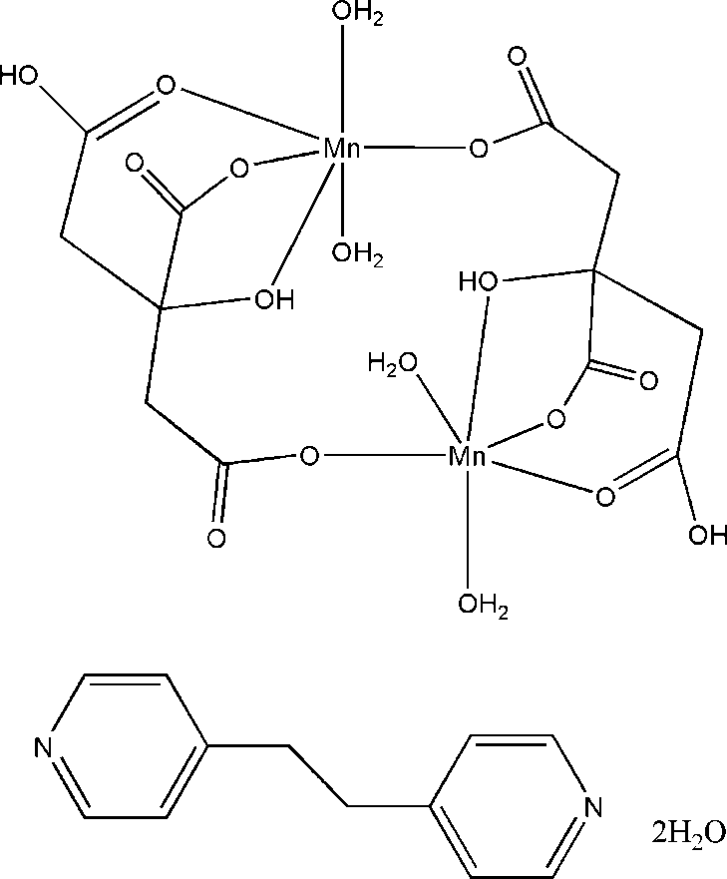


## Experimental

### 

#### Crystal data

[Mn_2_(C_6_H_6_O_7_)_2_(H_2_O)_4_]·C_12_H_12_N_2_·2H_2_O
*M* *_r_* = 782.43
Triclinic, 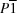

*a* = 9.3950 (19) Å
*b* = 9.5880 (19) Å
*c* = 10.252 (2) Å
α = 68.90 (3)°
β = 67.74 (3)°
γ = 78.16 (3)°
*V* = 794.8 (3) Å^3^
*Z* = 1
Mo *K*α radiation
μ = 0.88 mm^−1^
*T* = 293 K
0.40 × 0.20 × 0.20 mm

#### Data collection

Bruker SMART CCD diffractometer
Absorption correction: multi-scan (*SADABS*; Bruker, 1997 ▶) *T*_min_ = 0.719, *T*_max_ = 0.843
4449 measured reflections
3048 independent reflections
2780 reflections with *I* > 2σ(*I*)
*R*_int_ = 0.014

#### Refinement

*R*[*F* ^2^ > 2σ(*F* ^2^)] = 0.033
*wR*(*F* ^2^) = 0.092
*S* = 1.05
3048 reflections
239 parameters
7 restraints
H atoms treated by a mixture of independent and constrained refinement
Δρ_max_ = 0.52 e Å^−3^
Δρ_min_ = −0.42 e Å^−3^

### 

Data collection: *SMART* (Bruker, 1997 ▶); cell refinement: *SAINT* (Bruker, 1997 ▶); data reduction: *SAINT*; program(s) used to solve structure: *SHELXS97* (Sheldrick, 2008 ▶); program(s) used to refine structure: *SHELXL97* (Sheldrick, 2008 ▶); molecular graphics: *SHELXTL* (Sheldrick, 2008 ▶); software used to prepare material for publication: *SHELXTL*.

## Supplementary Material

Crystal structure: contains datablock(s) I, global. DOI: 10.1107/S1600536812032771/bx2421sup1.cif

Structure factors: contains datablock(s) I. DOI: 10.1107/S1600536812032771/bx2421Isup2.hkl

Additional supplementary materials:  crystallographic information; 3D view; checkCIF report

## Figures and Tables

**Table 1:** Hydrogen-bond geometry (Å, °)

*D*—H⋯*A*	*D*—H	H⋯*A*	*D*⋯*A*	*D*—H⋯*A*
O1*W*—H1*WA*⋯O3	0.96 (1)	1.91 (1)	2.869 (3)	175 (3)
O1—H1*O*⋯O6	0.93 (1)	1.74 (1)	2.6020 (19)	153 (2)
O1*W*—H1*WB*⋯O7^i^	0.96 (1)	2.02 (2)	2.903 (3)	152 (3)
O5—H5⋯N11^ii^	0.82	1.84	2.649 (2)	171
O8—H8*A*⋯O5^ii^	0.86 (1)	1.84 (1)	2.694 (2)	170 (2)
O8—H8*B*⋯O7^iii^	0.86 (1)	2.06 (1)	2.872 (2)	158 (2)
O8—H8*B*⋯O7^iv^	0.86 (1)	2.56 (2)	3.075 (3)	119 (2)
O9—H9*A*⋯O1*W* ^iii^	0.86 (1)	1.88 (1)	2.722 (3)	168 (3)
O9—H9*B*⋯O2^v^	0.86 (1)	1.97 (1)	2.829 (2)	173 (3)

Symmetry codes: (i) 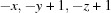; (ii) 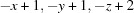; (iii) 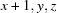; (iv) 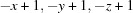; (v) 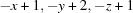.

**Fig. 1.**
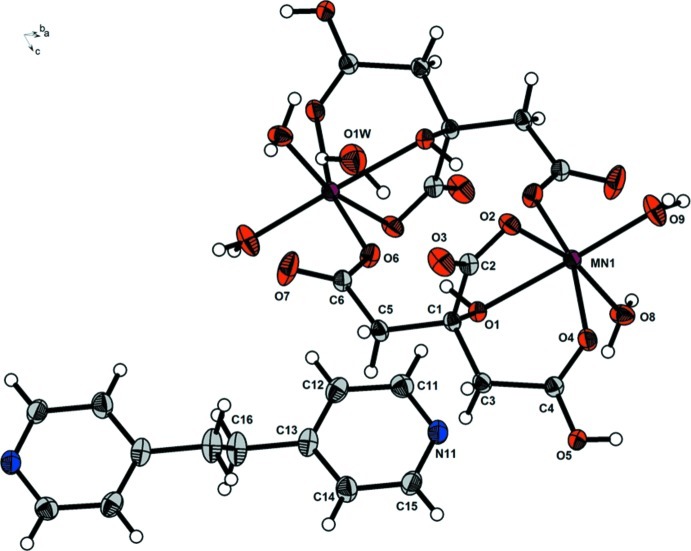
The structure of the title compound showing the atom numbering scheme. Displacement ellipsoids are shown at the 50% probability level. The labelled atoms are related with unlabelled atoms by symmetry code: [1-x, 1-y, 1-z] for diaqua- bis(citrato)di-manganese(II) fragment and [-x, -y, 2-z ] for 1,2-bis(pyridin-4-yl)ethane solvate.

## supplementary crystallographic information

### Comment

As models to examine the interaction between transition metal ions with biologically active molecules (Daniele, *et al.*, 2008; Parkin, 2004; Tshuva & Lippard, 2004; Stoumpos, *et al.*, 2009), we have intensively studied the interaction of the transition metal ions with the various acids such as benzoic acid, citric acid, and amino acids. Therefore, we have reported a variety of structures of copper(II), cadmium(II), nickel(II), cobalt(II), and zinc(II) benzoates with quinoxaline, 6-methylquinoline, 3-methylquinoline, *trans*-1-(2-pyridyl)-2-(pyridin-4-yl)ethylene, and di-2-pyridyl ketone (Lee, *et al.*, 2008; Yu, *et al.*, 2008; Park, *et al.*, 2008; Shin, *et al.*, 2009; Song, *et al.*, 2009; Yu, *et al.*, 2008, 2009; Kim, *et al.*, 2011). However, manganese as a metal ion source was rarely used. In this work, we have employed manganese(II) nitrate as a building block and citric acid as a ligand. We report here on the structure of new tetraaquadicitratodimanganese(II)-1,2-bis(pyridin-4-yl)ethane-dihydrate, [Mn_2_(H_2_O)_4_(C_6_H_8_O_7_)_2_].(C_12_H_12_N_2_).2(H_2_O). The molecular structure of the title compound is shown in Fig. 1. The asymmetric unit of the title compound, C_24_H_36_Mn_2_N_2_O_20_ , comprises half of a centrosymmetric dimer , half of a 1,2-bis(pyridin-4-yl)ethane ligand and one water molecule. Two citrate ligands bridge two Mn^II^ ions, and each Mn^II^ is coordinated by four oxygen atoms from the citrates ligand and two water oxygen atoms, forming a distorted octahedral environment. In the crystal, O—H···O hydrogen bonds link the cetrosymmetric dimer and free H_2_O components into a three-dimensional structure. The crystal structure is further stabilized by intermolecular π-π interactions [centroid = C11–C15/N11; centroid–centroid distance = 3.792 (2) Å symmetry code: 1-x, -y, 2-z ].

### Experimental

Citric acid (19.4 mg,0.1 mmol) and Mn(NO_3_)_2_.H_2_O(18.3 mg, 0.1 mmol) were dissolved in 4 ml H_2_O and carefully layered by 4 ml acetonitrile solution of 1,2-bis(pyridin-4-yl)ethane (38.0 mg, 0.2 mmol). Suitable crystals of the title compound were obtained in a month.

### Refinement

H atoms bonded to C atoms were placed in calculated positions with C—H distances of 0.93 Å for aromatic C atoms and 0.97 Å for methylene C atoms. They were included in the refinement in riding-motion approximation with *U*_iso_(H) = 1.2*U*_eq_(C). H atom bonded to O atom was placed in the calculated position with O—H distance of 0.82 Å for carboxylate O atom, and it was included in the refinement in riding-motion approximation with *U*_iso_(H) = 1.5*U*_eq_(C). The position of O—H atom of the hydroxyl group was refined with O—H = 0.93 Å and *U*_iso_(H) = 1.5*U*_eq_(N). The positions of O—H atoms of the coordinated water ligands were refined with O—H = 0.86 Å and *U*_iso_(H) = 1.2*U*_eq_(N). The positions of O—H atoms of the free water molecule were refined with O—H = 0.96 Å and *U*_iso_(H) = 1.2*U*_eq_(N).

### Crystal data

**Table:**

[Mn_2_(C_6_H_6_O_7_)_2_(H_2_O)_4_]·C_12_H_12_N_2_·2H_2_O	*Z* = 1
*M**_r_* = 782.43	*F*(000) = 404
Triclinic, *P*1	*D*_x_ = 1.635 Mg m^−^^3^
Hall symbol: -P 1	Mo *K*α radiation, λ = 0.71073 Å
*a* = 9.3950 (19) Å	Cell parameters from 11909 reflections
*b* = 9.5880 (19) Å	θ = 2.7–27.6°
*c* = 10.252 (2) Å	µ = 0.88 mm^−^^1^
α = 68.90 (3)°	*T* = 293 K
β = 67.74 (3)°	Block, colourless
γ = 78.16 (3)°	0.40 × 0.20 × 0.20 mm
*V* = 794.8 (3) Å^3^	

### Data collection

**Table:**

Bruker SMART CCD diffractometer	3048 independent reflections
Radiation source: fine-focus sealed tube	2780 reflections with *I* > 2σ(*I*)
Graphite monochromator	*R*_int_ = 0.014
φ and ω scans	θ_max_ = 26.0°, θ_min_ = 2.3°
Absorption correction: multi-scan (*SADABS*; Bruker, 1997)	*h* = −11→11
*T*_min_ = 0.719, *T*_max_ = 0.843	*k* = −8→11
4449 measured reflections	*l* = −12→11

### Refinement

**Table:**

Refinement on *F*^2^	Primary atom site location: structure-invariant direct methods
Least-squares matrix: full	Secondary atom site location: difference Fourier map
*R*[*F*^2^ > 2σ(*F*^2^)] = 0.033	Hydrogen site location: inferred from neighbouring sites
*wR*(*F*^2^) = 0.092	H atoms treated by a mixture of independent and constrained refinement
*S* = 1.05	*w* = 1/[σ^2^(*F*_o_^2^) + (0.0603*P*)^2^ + 0.2356*P*] where *P* = (*F*_o_^2^ + 2*F*_c_^2^)/3
3048 reflections	(Δ/σ)_max_ = 0.001
239 parameters	Δρ_max_ = 0.52 e Å^−^^3^
7 restraints	Δρ_min_ = −0.42 e Å^−^^3^

### Special details

**Table:**

Geometry. All e.s.d.'s (except the e.s.d. in the dihedral angle between two l.s. planes) are estimated using the full covariance matrix. The cell e.s.d.'s are taken into account individually in the estimation of e.s.d.'s in distances, angles and torsion angles; correlations between e.s.d.'s in cell parameters are only used when they are defined by crystal symmetry. An approximate (isotropic) treatment of cell e.s.d.'s is used for estimating e.s.d.'s involving l.s. planes.
Refinement. Refinement of *F*^2^ against ALL reflections. The weighted *R*-factor *wR* and goodness of fit *S* are based on *F*^2^, conventional *R*-factors *R* are based on *F*, with *F* set to zero for negative *F*^2^. The threshold expression of *F*^2^ > σ(*F*^2^) is used only for calculating *R*-factors(gt) *etc*. and is not relevant to the choice of reflections for refinement. *R*-factors based on *F*^2^ are statistically about twice as large as those based on *F*, and *R*- factors based on ALL data will be even larger.

### Fractional atomic coordinates and isotropic or equivalent isotropic displacement parameters (Å^2^)

**Table:**

	*x*	*y*	*z*	*U*_iso_*/*U*_eq_	
Mn1	0.59630 (3)	0.69243 (3)	0.60317 (3)	0.02605 (12)	
O1	0.42810 (14)	0.50818 (14)	0.71524 (14)	0.0253 (3)	
H1O	0.422 (3)	0.467 (2)	0.648 (2)	0.038*	
O2	0.38654 (15)	0.79182 (15)	0.54871 (15)	0.0344 (3)	
O3	0.13155 (17)	0.80099 (18)	0.6601 (2)	0.0491 (4)	
O4	0.47047 (16)	0.73580 (18)	0.81310 (15)	0.0377 (3)	
O5	0.34758 (17)	0.66928 (18)	1.05400 (15)	0.0400 (4)	
H5	0.4213	0.7042	1.0522	0.060*	
O6	0.31979 (15)	0.40470 (17)	0.57203 (15)	0.0355 (3)	
O7	0.06796 (18)	0.4110 (3)	0.6235 (2)	0.0632 (6)	
O8	0.76699 (16)	0.56448 (18)	0.70557 (16)	0.0389 (3)	
H8A	0.738 (3)	0.4919 (19)	0.7866 (15)	0.047*	
H8B	0.8528 (15)	0.525 (3)	0.659 (2)	0.047*	
O9	0.70528 (19)	0.89824 (18)	0.5069 (2)	0.0498 (4)	
H9A	0.8044 (3)	0.892 (3)	0.476 (3)	0.060*	
H9B	0.672 (3)	0.9906 (10)	0.498 (3)	0.060*	
C1	0.27525 (19)	0.5802 (2)	0.76103 (19)	0.0243 (4)	
C2	0.2623 (2)	0.7377 (2)	0.6467 (2)	0.0289 (4)	
C3	0.2476 (2)	0.5940 (2)	0.9129 (2)	0.0294 (4)	
H3A	0.1475	0.6478	0.9430	0.035*	
H3B	0.2421	0.4939	0.9836	0.035*	
C4	0.3654 (2)	0.6717 (2)	0.9252 (2)	0.0277 (4)	
C5	0.1528 (2)	0.4839 (2)	0.7782 (2)	0.0285 (4)	
H5A	0.1468	0.3963	0.8648	0.034*	
H5B	0.0535	0.5413	0.7976	0.034*	
C6	0.1791 (2)	0.4310 (2)	0.6473 (2)	0.0295 (4)	
N11	0.4324 (2)	0.18749 (19)	0.9584 (2)	0.0367 (4)	
C11	0.4410 (3)	0.1513 (2)	0.8411 (2)	0.0392 (5)	
H11	0.5245	0.1776	0.7540	0.047*	
C12	0.3275 (3)	0.0755 (2)	0.8483 (3)	0.0401 (5)	
H12	0.3343	0.0505	0.7664	0.048*	
C13	0.2026 (2)	0.0363 (2)	0.9784 (3)	0.0397 (5)	
C14	0.1958 (3)	0.0774 (3)	1.0979 (3)	0.0433 (5)	
H14	0.1126	0.0544	1.1857	0.052*	
C15	0.3128 (3)	0.1524 (3)	1.0851 (3)	0.0408 (5)	
H15	0.3090	0.1790	1.1652	0.049*	
C16	0.0763 (3)	−0.0475 (3)	0.9912 (3)	0.0521 (6)	
H16A	0.0643	−0.1376	1.0761	0.062*	
H16B	0.1053	−0.0777	0.9032	0.062*	
O1W	0.0166 (2)	0.8352 (2)	0.4269 (2)	0.0644 (5)	
H1WA	0.061 (3)	0.824 (4)	0.501 (3)	0.077*	
H1WB	0.023 (4)	0.7418 (18)	0.410 (4)	0.077*	

### Atomic displacement parameters (Å^2^)

**Table:**

	*U*^11^	*U*^22^	*U*^33^	*U*^12^	*U*^13^	*U*^23^
Mn1	0.02480 (17)	0.02764 (18)	0.02587 (17)	−0.00515 (11)	−0.00565 (12)	−0.00986 (12)
O1	0.0215 (6)	0.0254 (6)	0.0297 (6)	0.0000 (5)	−0.0076 (5)	−0.0114 (5)
O2	0.0298 (7)	0.0293 (7)	0.0357 (7)	−0.0044 (6)	−0.0089 (6)	−0.0016 (6)
O3	0.0295 (8)	0.0379 (9)	0.0649 (11)	0.0061 (6)	−0.0130 (7)	−0.0066 (8)
O4	0.0375 (8)	0.0463 (8)	0.0320 (7)	−0.0179 (6)	−0.0006 (6)	−0.0189 (6)
O5	0.0428 (8)	0.0539 (9)	0.0291 (7)	−0.0222 (7)	−0.0092 (6)	−0.0126 (7)
O6	0.0265 (7)	0.0492 (9)	0.0378 (7)	−0.0015 (6)	−0.0071 (6)	−0.0263 (7)
O7	0.0296 (8)	0.1139 (16)	0.0738 (12)	−0.0066 (9)	−0.0134 (8)	−0.0644 (12)
O8	0.0288 (7)	0.0465 (9)	0.0312 (7)	−0.0008 (6)	−0.0072 (6)	−0.0048 (7)
O9	0.0355 (8)	0.0282 (8)	0.0753 (12)	−0.0089 (7)	−0.0104 (8)	−0.0094 (8)
C1	0.0204 (8)	0.0253 (9)	0.0265 (8)	−0.0026 (7)	−0.0042 (7)	−0.0108 (7)
C2	0.0267 (9)	0.0263 (9)	0.0346 (10)	−0.0004 (7)	−0.0106 (8)	−0.0110 (8)
C3	0.0294 (9)	0.0308 (10)	0.0273 (9)	−0.0082 (8)	−0.0032 (7)	−0.0119 (8)
C4	0.0281 (9)	0.0262 (9)	0.0297 (9)	−0.0019 (7)	−0.0078 (7)	−0.0121 (8)
C5	0.0223 (8)	0.0310 (10)	0.0317 (9)	−0.0056 (7)	−0.0032 (7)	−0.0133 (8)
C6	0.0261 (9)	0.0318 (10)	0.0328 (9)	−0.0038 (7)	−0.0083 (7)	−0.0135 (8)
N11	0.0367 (9)	0.0311 (9)	0.0455 (10)	−0.0044 (7)	−0.0202 (8)	−0.0078 (8)
C11	0.0400 (11)	0.0335 (11)	0.0415 (11)	−0.0017 (9)	−0.0152 (9)	−0.0077 (9)
C12	0.0450 (12)	0.0367 (11)	0.0487 (12)	0.0040 (9)	−0.0244 (10)	−0.0193 (10)
C13	0.0350 (11)	0.0345 (11)	0.0600 (14)	0.0020 (9)	−0.0235 (10)	−0.0209 (10)
C14	0.0341 (11)	0.0492 (13)	0.0496 (13)	−0.0077 (10)	−0.0124 (10)	−0.0180 (11)
C15	0.0446 (12)	0.0429 (12)	0.0438 (12)	−0.0066 (10)	−0.0208 (10)	−0.0154 (10)
C16	0.0394 (13)	0.0454 (14)	0.0871 (19)	0.0007 (11)	−0.0266 (13)	−0.0350 (13)
O1W	0.0568 (11)	0.0585 (12)	0.0768 (13)	−0.0075 (9)	−0.0278 (10)	−0.0126 (11)

### Geometric parameters (Å, º)

**Table:**

Mn1—O6^i^	2.1319 (15)	C3—C4	1.518 (3)
Mn1—O9	2.1395 (17)	C3—H3A	0.9700
Mn1—O4	2.1720 (15)	C3—H3B	0.9700
Mn1—O8	2.1725 (16)	C5—C6	1.519 (3)
Mn1—O2	2.1871 (15)	C5—H5A	0.9700
Mn1—O1	2.2905 (16)	C5—H5B	0.9700
O1—C1	1.440 (2)	N11—C11	1.337 (3)
O1—H1O	0.930 (2)	N11—C15	1.341 (3)
O2—C2	1.275 (2)	C11—C12	1.375 (3)
O3—C2	1.234 (2)	C11—H11	0.9300
O4—C4	1.246 (2)	C12—C13	1.389 (3)
O5—C4	1.259 (2)	C12—H12	0.9300
O5—H5	0.8200	C13—C14	1.392 (3)
O6—C6	1.280 (2)	C13—C16	1.506 (3)
O6—Mn1^i^	2.1319 (15)	C14—C15	1.375 (3)
O7—C6	1.223 (2)	C14—H14	0.9300
O8—H8A	0.860 (2)	C15—H15	0.9300
O8—H8B	0.859 (2)	C16—C16^ii^	1.518 (5)
O9—H9A	0.860 (2)	C16—H16A	0.9700
O9—H9B	0.860 (2)	C16—H16B	0.9700
C1—C3	1.529 (3)	O1W—H1WA	0.960 (2)
C1—C5	1.540 (2)	O1W—H1WB	0.959 (2)
C1—C2	1.558 (3)		
			
O6^i^—Mn1—O9	103.57 (7)	C1—C3—H3A	108.0
O6^i^—Mn1—O4	163.30 (6)	C4—C3—H3B	108.0
O9—Mn1—O4	93.05 (7)	C1—C3—H3B	108.0
O6^i^—Mn1—O8	93.96 (6)	H3A—C3—H3B	107.2
O9—Mn1—O8	95.36 (7)	O4—C4—O5	122.97 (17)
O4—Mn1—O8	85.97 (6)	O4—C4—C3	121.25 (16)
O6^i^—Mn1—O2	92.00 (6)	O5—C4—C3	115.76 (16)
O9—Mn1—O2	94.89 (6)	C6—C5—C1	116.30 (15)
O4—Mn1—O2	84.89 (6)	C6—C5—H5A	108.2
O8—Mn1—O2	166.60 (5)	C1—C5—H5A	108.2
O6^i^—Mn1—O1	83.80 (6)	C6—C5—H5B	108.2
O9—Mn1—O1	166.66 (6)	C1—C5—H5B	108.2
O4—Mn1—O1	79.57 (6)	H5A—C5—H5B	107.4
O8—Mn1—O1	95.19 (6)	O7—C6—O6	124.37 (18)
O2—Mn1—O1	73.53 (5)	O7—C6—C5	119.40 (17)
C1—O1—Mn1	107.05 (10)	O6—C6—C5	116.17 (16)
C1—O1—H1O	101.9 (14)	C11—N11—C15	121.11 (18)
Mn1—O1—H1O	112.3 (14)	N11—C11—C12	120.5 (2)
C2—O2—Mn1	114.86 (12)	N11—C11—H11	119.7
C4—O4—Mn1	132.39 (13)	C12—C11—H11	119.7
C4—O5—H5	109.5	C11—C12—C13	119.8 (2)
C6—O6—Mn1^i^	127.51 (12)	C11—C12—H12	120.1
Mn1—O8—H8A	118.4 (17)	C13—C12—H12	120.1
Mn1—O8—H8B	123.3 (17)	C12—C13—C14	118.3 (2)
H8A—O8—H8B	101 (2)	C12—C13—C16	121.5 (2)
Mn1—O9—H9A	117.2 (19)	C14—C13—C16	120.2 (2)
Mn1—O9—H9B	134 (2)	C15—C14—C13	119.5 (2)
H9A—O9—H9B	109 (3)	C15—C14—H14	120.2
O1—C1—C3	106.79 (14)	C13—C14—H14	120.2
O1—C1—C5	110.98 (14)	N11—C15—C14	120.7 (2)
C3—C1—C5	108.23 (15)	N11—C15—H15	119.7
O1—C1—C2	110.46 (14)	C14—C15—H15	119.7
C3—C1—C2	110.69 (15)	C13—C16—C16^ii^	111.7 (2)
C5—C1—C2	109.64 (15)	C13—C16—H16A	109.3
O3—C2—O2	125.25 (18)	C16^ii^—C16—H16A	109.3
O3—C2—C1	116.81 (17)	C13—C16—H16B	109.3
O2—C2—C1	117.94 (16)	C16^ii^—C16—H16B	109.3
C4—C3—C1	117.19 (15)	H16A—C16—H16B	107.9
C4—C3—H3A	108.0	H1WA—O1W—H1WB	111 (3)
			
O6^i^—Mn1—O1—C1	−128.46 (11)	C5—C1—C2—O2	−134.76 (17)
O9—Mn1—O1—C1	−4.0 (3)	O1—C1—C3—C4	54.5 (2)
O4—Mn1—O1—C1	53.18 (11)	C5—C1—C3—C4	174.00 (15)
O8—Mn1—O1—C1	138.10 (11)	C2—C1—C3—C4	−65.8 (2)
O2—Mn1—O1—C1	−34.53 (10)	Mn1—O4—C4—O5	150.23 (16)
O6^i^—Mn1—O2—C2	113.17 (14)	Mn1—O4—C4—C3	−31.3 (3)
O9—Mn1—O2—C2	−143.02 (14)	C1—C3—C4—O4	7.7 (3)
O4—Mn1—O2—C2	−50.38 (14)	C1—C3—C4—O5	−173.68 (17)
O8—Mn1—O2—C2	−3.2 (3)	O1—C1—C5—C6	−51.4 (2)
O1—Mn1—O2—C2	30.23 (13)	C3—C1—C5—C6	−168.30 (16)
O6^i^—Mn1—O4—C4	−3.7 (3)	C2—C1—C5—C6	70.9 (2)
O9—Mn1—O4—C4	170.83 (19)	Mn1^i^—O6—C6—O7	−4.3 (3)
O8—Mn1—O4—C4	−94.01 (19)	Mn1^i^—O6—C6—C5	172.89 (12)
O2—Mn1—O4—C4	76.19 (19)	C1—C5—C6—O7	−150.0 (2)
O1—Mn1—O4—C4	2.03 (18)	C1—C5—C6—O6	32.6 (3)
Mn1—O1—C1—C3	−85.32 (13)	C15—N11—C11—C12	−0.6 (3)
Mn1—O1—C1—C5	156.92 (12)	N11—C11—C12—C13	0.1 (3)
Mn1—O1—C1—C2	35.10 (15)	C11—C12—C13—C14	0.8 (3)
Mn1—O2—C2—O3	158.86 (17)	C11—C12—C13—C16	−179.6 (2)
Mn1—O2—C2—C1	−20.1 (2)	C12—C13—C14—C15	−1.2 (3)
O1—C1—C2—O3	168.78 (17)	C16—C13—C14—C15	179.2 (2)
C3—C1—C2—O3	−73.2 (2)	C11—N11—C15—C14	0.2 (3)
C5—C1—C2—O3	46.2 (2)	C13—C14—C15—N11	0.7 (4)
O1—C1—C2—O2	−12.2 (2)	C12—C13—C16—C16^ii^	−113.7 (3)
C3—C1—C2—O2	105.91 (19)	C14—C13—C16—C16^ii^	65.8 (4)

Symmetry codes: (i) −*x*+1, −*y*+1, −*z*+1; (ii) −*x*, −*y*, −*z*+2.

### Hydrogen-bond geometry (Å, º)

**Table:**

*D*—H···*A*	*D*—H	H···*A*	*D*···*A*	*D*—H···*A*
O1*W*—H1*WA*···O3	0.96 (1)	1.91 (1)	2.869 (3)	175 (3)
O1—H1*O*···O6	0.93 (1)	1.74 (1)	2.6020 (19)	153 (2)
O1*W*—H1*WB*···O7^iii^	0.96 (1)	2.02 (2)	2.903 (3)	152 (3)
O5—H5···N11^iv^	0.82	1.84	2.649 (2)	171
O8—H8*A*···O5^iv^	0.86 (1)	1.84 (1)	2.694 (2)	170 (2)
O8—H8*B*···O7^v^	0.86 (1)	2.06 (1)	2.872 (2)	158 (2)
O8—H8*B*···O7^i^	0.86 (1)	2.56 (2)	3.075 (3)	119 (2)
O9—H9*A*···O1*W*^v^	0.86 (1)	1.88 (1)	2.722 (3)	168 (3)
O9—H9*B*···O2^vi^	0.86 (1)	1.97 (1)	2.829 (2)	173 (3)
C5—H5*B*···O3	0.97	2.48	2.834 (3)	101
C14—H14···O1*W*^vii^	0.93	2.59	3.357 (4)	141
C15—H15···O4^iv^	0.93	2.49	3.114 (3)	125

Symmetry codes: (i) −*x*+1, −*y*+1, −*z*+1; (iii) −*x*, −*y*+1, −*z*+1; (iv) −*x*+1, −*y*+1, −*z*+2; (v) *x*+1, *y*, *z*; (vi) −*x*+1, −*y*+2, −*z*+1; (vii) *x*, *y*−1, *z*+1.

## References

[bb1] Bruker (1997). *SMART*, *SAINT* and *SADABS* Bruker AXS Inc., Madison, Wisconsin, USA.

[bb2] Daniele, P. G., Foti, C., Gianguzza, A., Prenesti, E. & Sammartano, S. (2008). *Coord. Chem. Rev.* **252**, 1093–1107.

[bb3] Kim, J. H., Kim, C. & Kim, Y. (2011). *Acta Cryst.* E**67**, m3–m4. 10.1107/S1600536810049457 PMC3050134 21522553

[bb4] Lee, E. Y., Park, B. K., Kim, C., Kim, S.-J. & Kim, Y. (2008). *Acta Cryst.* E**64**, m286. 10.1107/S1600536807067876 PMC2960297 21201265

[bb5] Park, B. K., Jang, K.-H., Kim, P.-G., Kim, C. & Kim, Y. (2008). *Acta Cryst.* E**64**, m1141. 10.1107/S1600536808024859 PMC2960550 21201597

[bb6] Parkin, G. (2004). *Chem. Rev.* **104**, 699–767. 10.1021/cr0206263 14871139

[bb7] Sheldrick, G. M. (2008). *Acta Cryst.* A**64**, 112–122. 10.1107/S0108767307043930 18156677

[bb8] Shin, D. H., Han, S.-H., Kim, P.-G., Kim, C. & Kim, Y. (2009). *Acta Cryst.* E**65**, m658–m659. 10.1107/S1600536809017772 PMC2969578 21583021

[bb9] Song, Y. J., Lee, S.-W., Jang, K. H., Kim, C. & Kim, Y. (2009). *Acta Cryst.* E**65**, m1495–m1496. 10.1107/S1600536809045048 PMC2972024 21578547

[bb10] Stoumpos, C. C., Gass, I. A., Milios, C. J., Lalioti, N., Terzis, A., Aromi, G., Teat, S. J., Brechin, E. K. & Perlepes, S. P. (2009). *Dalton Trans.* pp. 307–317. 10.1039/b810835h 19089012

[bb11] Tshuva, E. Y. & Lippard, S. J. (2004). *Chem. Rev.* **104**, 987–1012. 10.1021/cr020622y 14871147

[bb12] Yu, S. M., Park, C.-H., Kim, P.-G., Kim, C. & Kim, Y. (2008). *Acta Cryst.* E**64**, m881–m882. 10.1107/S1600536808016516 PMC2961840 21202752

[bb13] Yu, S. M., Shin, D. H., Kim, P.-G., Kim, C. & Kim, Y. (2009). *Acta Cryst.* E**65**, m1045–m1046. 10.1107/S1600536809030281 PMC2969950 21577407

